# Aggressive Neuroblastoma in a Pediatric Patient with Severe Hemophilia A

**DOI:** 10.3390/pediatric13010018

**Published:** 2021-03-08

**Authors:** Lidia Costa, Maria Eduarda Couto, Juliana Moutinho, Ana Maia Ferreira, Emilia Costa, Susana Roncon, Luisa Lopes Santos, Eugenia Cruz, Sara Morais

**Affiliations:** 1 Centro de Coagulopatias Congénitas, Centro Hospitalar Universitário do Porto (CHUP), 4050-342 Porto, Portugal; u12084@chporto.min-saude.pt (L.C.); ecruz@ibmc.up.pt (E.C.); 2 Serviço de Onco-Hematologia, Instituto Português de Oncologia do Porto (IPO-Porto), 4200-072 Porto, Portugal; eduarda.scouto@gmail.com; 3 Serviço de Imuno-Hemoterapia, Instituto Português de Oncolologia do Porto (IPO-Porto), 4200-072 Porto, Portugal; juliana.moutinho@gmail.com (J.M.); mlsantos@ipoporto.min-saude.pt (L.L.S.); 4 Serviço de Pediatria, Instituto Português de Oncolologia do Porto (IPO-Porto), 4200-072 Porto, Portugal; ana.ferreira@ipoporto.min-saude.pt; 5 Unidade de Hematologia Pediátrica, Centro Materno-Infantil do Norte (CMIN), Centro Hospitalar Universitário do Porto (CHUP), 4050-651 Porto, Portugal; emvcosta@hotmail.com; 6 Serviço de Terapia Celular, Instituto Português de Oncolologia do Porto (IPO-Porto), 4200-072 Porto, Portugal; sroncon@ipoporto.min-saude.pt; 7 i3S-Basic&Clinical Research on Iron Biology (BCRIB), Instituto de Investigação e Inovação em Saúde, Universidade do Porto, 4200-135 Porto, Portugal; 8 Unidade Multidisciplinar de Investigação Biomédica, Instituto de Ciências Biomédicas, Universidade do Porto (UMIB/ICBAS/UP), 4050-313 Porto, Portugal

**Keywords:** hemophilia A, neuroblastoma, peripheral blood stem cell transplantation, prophylaxis

## Abstract

Despite the extensive information regarding hemophilia’s hemorrhagic complications, the literature on cancer in hemophilia is scarce, especially in pediatric patients. Many uncertainties remain concerning diagnosis and workup. We report a rare case of two severe diseases (neuroblastoma and hemophilia A (HA)) concomitantly present in the same pediatric patient. We highlight that the diagnosis of severe HA did not have a negative impact on the patient’s oncologic course. This case also illustrates the significance of the cooperation among different specialties and hospitals when caring for the same patient.

## 1. Introduction

Hemophilia A (HA) is a rare X-linked inherited bleeding disorder characterized by deficiency of FVIII with an estimated incidence of 1 in 5000 male births [1,2]. In patients with severe disease (FVIII levels < 0.01 IU/mL), spontaneous bleeding or hemorrhage after minimal trauma in joints or muscles are common. Replacement therapy with FVIII concentrates (prophylaxis) is the mainstay of treatment in hemophilia, mainly to prevent bleeding.

Neuroblastoma is the most common extracranial solid tumor in childhood [3], with a median age at diagnosis of 18 months [4] and having a heterogeneous clinical presentation. It is still unclear if hemophilia A (HA) is a predisposing condition to malignancy or if it conditions a worse prognosis in HA patients with neoplastic disorders.

Here, we describe a case of severe HA in a child with aggressive neuroblastoma, the first report in the literature to the best of our knowledge, which makes these findings rare and valuable.

## 2. Results

The patient was a black, male, full-term neonate from an uneventful pregnancy, unremarkable family history, unrelated parents, and born from routine eutocic delivery. At day 2 of life, several hematomas (extensive subgaleal hematoma, subdural small volume deposits, and subarachnoid blood without intraventricular bleeding) and severe secondary anemia (hemoglobin [Hb]: 6.6 g/dL) were diagnosed and he was admitted to the neonatal intensive care unit. Severe HA was diagnosed on day 3 of life [5]. Replacement therapy with recombinant FVIII concentrate (rFVIII) was started at a dose of 65 IU/kg every 8 h the first day, every 12 h the second and third days, and daily from the 6th day onward. On day 11 of life, he was discharged and FVIII infusion was stopped. He maintained slow reabsorption left parietal cephalohematoma. No inhibitors were detected.

The patient started regular follow-up at a hemophilia center. Genetic studies revealed point mutation c4969C > T p.(Gln1657*) on exon 14 of the *F8* gene in the child. The infant presented normal development without relevant incidents. During the first 18 months of life, he underwent three on-demand treatments with rFVIII due to minor or moderate bleedings (no hemarthrosis).

**Figure 1:**
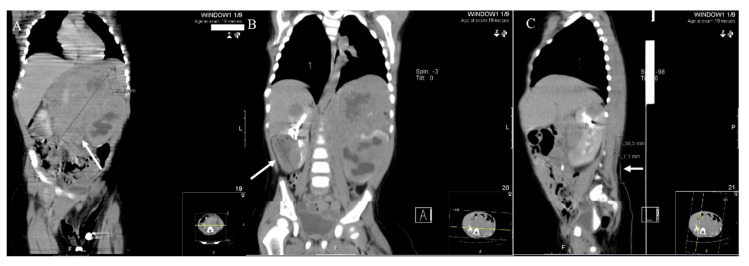
Thoracic, abdominal, and pelvic CT scan performed at diagnosis: (**A**) large described mass over left kidney (arrow); (**B**) metastatic lesion on right kidney (arrow); (**C**) hemorrhagic infiltration of the paravertebral muscles (arrowhead).

At 19 months of age, the child was admitted at the emergency department due to loss of weight (15% of body weight in 43 days), lethargy, somnolence, anorexia, and discomfort. Physical exam showed pallor and right paravertebral mass, suggesting paravertebral muscle hematoma (confirmed with ultrasound). The initial laboratorial workup unveiled mild anemia (Hb: 9.7 g/dL, age-adjusted reference range (RR): 10.5–13.5 g/dL), mild reactive thrombocytosis (platelets: 445 × 10^9^/L, RR: 150–400 × 10^9^/L), and increased lactate dehydrogenase (LDH; 1481 IU/L, RR: 130–350 IU/L) and uric acid (8.9 mg/dL, RR: 2.0–5.5 mg/dL). The thoracoabdominal–pelvic CT scan (Figure 1) showed a large retroperitoneal solid mass in the left quadrants (larger diameter of 12.8 × 10.8 × 8 cm) with areas of necrosis and calcification involving the left kidney. It also presented hydronephrosis. The right kidney had two solid masses measuring 3 and 5 cm each, probable metastatic lesions. No adenopathies were found. The paravertebral muscles were bilaterally thick (approximately 57 × 7 mm), suggesting hemorrhagic infiltration. Due to the patient’s age, location, clinical presentation, and imaging findings, it was assumed that neuroblastoma would be a likely diagnosis.

The patient was admitted to the pediatric ward (day 0). He immediately started replacement therapy with rFVIII (50 IU/kg) due to paravertebral hematomas with good response. This was followed by the initiation of prophylaxis with rFVIII 25 IU/kg, three times per week. He had developed spontaneous tumor lysis syndrome secondary to a disseminated neuroblastoma de novo, with left hydronephrosis.

The infant was transferred into the pediatric intensive care unit (ICU). As relevant complications, he developed mild hypertension after surgical biopsy (at day + 6), promptly corrected, anemia (Hb: 7.3 g/dL) with the need for packed red blood cells transfusion, and low-grade fever paired with elevated C reactive protein. Despite no pathogenic agents being isolated, he completed one cycle of empiric antibiotic therapy (piperacillin/tazobactam).

The laboratory workup (urinary catecholamines, histology of the fragment, immunophenotyped, and genetic profile) confirmed neuroblastoma diagnosis. The genetic tests demonstrated relative loss of 1p36 and relative gain of *MYCN*. The patient was diagnosed with undifferentiated large cells neuroblastoma, stage IV International Neuroblastoma Staging System (INSS), due to ganglionic regions, bone marrow, and both kidneys’ involvement.

At day + 15, the patient was directly referred to an oncologic facility. He began chemotherapy: the rapid-COJEC protocol (cisplatin, vincristine, carboplatin, etoposide, and cyclophosphamide). The first cycle was complicated by mild hypertension (needing a three-drug scheduled regimen to achieve blood pressure control) and *Clostridium difficile* infection (treated with metronidazole).

A metaiodobenzylguanidine (MIBG) and CT scan carried out four months later showed only partial response of the primary tumor, with persistence of the smaller metastatic renal lesions. Therefore, he started second line therapy. The treatment consisted of five courses of chemotherapy (TVD: topotecan, vincristine, and doxorubicin). Peripheral blood mobilized stem cells were collected for an autologous stem cell transplant (ASCT) to obtain a tandem transplant after myeloablative therapy (MAT) was planned, following the protocol of Park et al. [2]. Surgery was performed between the third and fourth TVD courses, allowing macroscopic complete removal of a left adrenal mass and biopsy of a small right kidney nodule (later histologically confirmed as metastatic). At this point, although the disease persisted, bone marrow aspiration and biopsy were both negative. A central venous catheter (CVC) was placed for the apheresis procedures.

The patient was subjected to third line therapy with the MATIN protocol (^131^I-mIBG (metaiodobenzylguanidine)/topotecan). The first ASCT was accomplished 10 months after diagnosis. The conditioning regimen consisted of busulfan plus melphalan. The second ASCT was performed two months later. Thirteen months after diagnosis, the MIBG scan was negative, urinary catecholamines were normal (dopamine, vanillylmandelic acid, and homovanillic acid), bone marrow aspirate and biopsy maintained negative, but the CT scan showed two persistent residual lesions in the right kidney (the same location as the previous metastasis).

He completed treatment with external beam radiation therapy toward the primary site and metastasis, followed by five courses of dinutuximab-beta alternated with six courses of oral isotretinoin.

The two residual renal nodules remained stable and there was no further evidence of neuroblastoma persistence. Presently, the patient is engaged in a clinical trial with oral difluoromethylornithine (DFMO).

Since the oncologic disease diagnosis, the patient remained on prophylaxis with rFVIII three times per week (25 IU/kg). Prophylaxis was adjusted to the different tumor treatment procedures, adapting FVIII doses to the procedures’ hemorrhagic risk (Table 1). During the aplasia period, he underwent daily prophylaxis. The patient did not experience major bleeding events, adverse reactions, increased use of packed red blood cells, or the development of FVIII inhibitors.

Currently, the child is under prophylaxis with an extended half-life (EHL) rFVIII factor, 25 IU/kg, twice per week.

## 3. Discussion

In infancy, abdominal masses should raise suspicion of four solid tumors: neuroblastoma, Wilms tumor, germ cells tumor, or soft tissue sarcoma [6].

Hemophilia A is currently not associated with oncologic diseases in childhood, except in immunocompromised patients [7], which was not the case here. There are few reports of oncologic diseases parallel to congenital hemophilia diagnosis [7], so clinical expertise in this setting is scarce. To date, there are no reports in the literature of neuroblastoma in a severe HA patient.

Our patient presented at cancer diagnosis without any previous hemarthrosis and a moderate bleeding phenotype (since the subgaleal hemorrhage at birth). The finding of the paravertebral muscle hematoma at the time of neuroblastoma detection motivated the beginning of primary prophylaxis.

In patients with severe HA, the differential diagnosis between hematomas in the retroperitoneal location and oncologic diseases is not always clear. In this case, signs of severe disease were promptly recognized at admission, namely in the blood chemistry, already suggesting an underlying aggressive disorder. A high clinical suspicion index was important for diagnosis as well as complete body imaging with a CT scan, which provided the distinction between oncologic disease and hematoma.

Beyond the differential diagnostic problems, enormous challenges are faced in the care of a cancer patient with an underlying severe bleeding disease. As highlighted by Franchini [8] whether patients with hemophilia and cancer present treatment limitations resulting in worse prognosis, such as therapy-related thrombocytopenia or mucosal bleeding, is unclear [8]. Thus, it is still unknown if they present a higher incidence of adverse hemorrhagic events due to their condition [8].

High-risk neuroblastoma can be refractory to conventional treatment approaches, requiring more aggressive strategies to achieve disease remission and improve outcome [3,4]. These include high-dose chemotherapy protocols that exceed bone marrow tolerance (MAT) followed by autologous peripheral blood stem cells (PBSC) collection and infusion [3,4,9], as described. Tandem ASCT presents an increased hemorrhagic risk [9], thus adding an additional complication to the patient’s management.

So, if managing a patient with severe HA initially appears to be a challenge, it only increases when associated with such an aggressive tumor. Severe HA presents with an augmented risk of spontaneous bleeding per se. Nevertheless, multiple therapies with high bleeding risk by themselves are also needed in the oncologic setting. Therefore, in our patient, these two concurrent entities were handled together, even though they both presented high bleeding risk. Hence, the patient was always successfully managed from the hematologists’ criteria with on-demand treatment or prophylaxis with rFVIII, without any impairment of the cancer treatment (Table 1).

## 4. Conclusions

An accurate hemorrhagic prophylaxis allowed the accomplishment of the entire oncologic therapeutic protocol with success. Hemophilia was neither a worse prognosis determinant nor an obstacle to the clinical approach. Our pediatric patient neither experienced severe bleeding events, adverse reactions, nor increased need for transfusion support. Despite having severe HA, this patient’s oncologic prognosis was not negatively affected.

Neuroblastoma is an aggressive disease, especially if metastases are already present at diagnosis. This is the first case reported in the literature of a neuroblastoma, a severe oncologic disease, along with severe HA. Close work between physicians from different hospitals dedicated to bleeding disorders and pediatric oncology was required for the successful management of the patient.

## Figures and Tables

**Table 1:** Hematological patient management (rFVIII regimen) during oncologic treatment (according to the protocol of Park et al. (2019).

FVIII Replacement Scheme	Major Hemorrhagic Procedures	Immediately Before	1st Day	2nd Day	8 Following Days
	(by Chronologic Order)				
Chemotherapy and invasive maneuvers	Standard chemotherapy	25 IU/kg 3×/week			
	(Rapid COJEC protocol + TVD cycles)				
	Retroperitoneal tumor excision	50 IU/kg	40 IU/kg		40 IU/lg q12 h
			q8 h		
	^131^I-mIBG + Topotecan	25 IU/kg 3×/week			
	Insertion/replacement	50 IU/kg	40 IU/kg	40 IU/kg q12 h	25 IU/kg 3×/week
	of CVC		q8 h		
	PBSC collection	50 IU/kg	50 IU/kg 1st dose + 40 UI/kg q8 h	25 IU/kg 3×/week	
	(5 day mobilization with G-CSF 12 µg/kg/day + leukapheresis) *				
Double autologous hematopoietic transplant	High-dose chemotherapy (busulfan + melphalan)	25 IU/kg 3×/week			
	PBSC infusion	25 IU/kg 3×/week	40 IU/kg q8 h		25 IU/kg q24 h **
	(3.5 × 10^6^ CD34 + cells/kg per transplant)				

Caption: * Platelet count > 50 × 10^9^/L in all conditions, except in leukapheresis (platelet count > 100 × 10^9^/L); ** 25 IU/kg daily during the aplasia period; COJEC: cisplatin, vincristine, carboplatin, etoposide, and cyclophosphamide; TVD: topotecan, vincristine, and doxorubicin; CVC: central venous catheter; G-CSF: granulocyte colony stimulating factor; ^131^I-mIBG: metaiodobenzylguanidine; PBSC: peripheral blood stem cells; rFVIII: recombinant factor FVIII.

## Data Availability

Not applicable.

## Abbreviations

ASCT	Autologous stem cell transplantion
COJEC	Cisplatin, vincristine, carboplatin, etoposide, cyclophosphamide
CT	Computed tomography
CVC	Central venous catheter
DMFO	Difluoromethylornithine
EHL	Extended half-life
FVIII	Factor VIII of the coagulation
G-CSF	Granulocyte colony stimulating factor
HA	Hemophilia A
Hb	Hemoglobin
ICU	Intensive care unit
^131^I-mIBG	Metaiodobenzylguanidine
INSS	International Neuroblastoma Staging System
LDH	Lactate dehydrogenase
MATIN	Metaiodobenzylguanidine, topotecan
PBSC	Peripheral blood stem cells
rFVIII	Recombinant FVIII concentrate
RR	Reference range
TVD	Topotecan, vincristine, doxorubicin
